# Access to Nature *via* Virtual Reality: A Mini-Review

**DOI:** 10.3389/fpsyg.2021.725288

**Published:** 2021-10-05

**Authors:** Hansen Li, Xing Zhang, Hongying Wang, Zongqian Yang, Haowei Liu, Yang Cao, Guodong Zhang

**Affiliations:** ^1^Key Laboratory of Physical Fitness Evaluation and Motor Function Monitoring of General Administration of Sports of China, Institute of Sports Science, College of Physical Education, Southwest University, Chongqing, China; ^2^Department of Basketball and Volleyball, Chengdu Sport University, Chengdu, China; ^3^College of Physical Education, JiMei University, Xiamen, China; ^4^Clinical Epidemiology and Biostatistics, School of Medical Sciences, Örebro University, Örebro, Sweden; ^5^Unit of Integrative Epidemiology, Institute of Environmental Medicine, Karolinska Institutet, Stockholm, Sweden

**Keywords:** nature exposure, virtual reality, mood, stress, health benefit, virtual environment

## Abstract

Nature exposure is known to promote physical and mental health. However, actual nature exposure may be difficult to achieve for the population of people with physical disabilities or chronic conditions. Therefore, many attempts have been made to duplicate nature exposure *via* media devices, and virtual reality (VR) is deemed as a promising technology due to its advantage in creating a sense of immersion. Generally, current studies suggest that being exposed to virtual nature may contribute to psychological and physiological relaxation. Besides, some pieces of evidence indicate that virtual nature may improve attentional resources, cognitive performance, and pain experience. Although VR is deemed as an advanced media, insufficient evidence was found concerning the advantages of VR over traditional two-dimensional media when it comes to simulated nature exposure. On the other hand, computer-generated (CG) scenarios were found to be more beneficial than 360° videos, and mini-games may be useful in creating an interactive VR format for simulated nature exposure. Further research is needed because of the limited relevant studies.

## Introduction

Being exposed to nature, including blue and green natural environments, is known to elicit a range of physical and mental health benefits (e.g., improve emotional states, reduce the risk of mental health issues, and benefit cardiovascular functions) (Gascon et al., 2015; White et al., 2021). Nowadays, various natural environments provide urban dwellers with important health resources to mitigate harmful exposures and their effects on urban living, such as pollution, attention depletion, and stressful short-term experiences (Ilies et al., 2007; Li H. et al., 2020). However, while human–nature interaction is encouraged, there is a considerable amount of people who have difficulties accessing ideal natural environments due to limited residential areas and physical conditions, especially for the elderly and patients with disabilities or chronic conditions (Browning et al., 2019). Therefore, there has been a growing interest in using virtual environments that depict nature to enhance public health (Reddon and Durante, 2018). In response, several studies aimed to duplicate nature exposure *via* traditional media, such as photos and videos (Jo et al., 2019). However, in many cases, only subtle benefits were obtained in indoor experiments, which might be due to the limited visual contact *via* traditional media (Jo et al., 2019).

On the other hand, virtual reality (VR) is an advanced media that can simulate highly realistic virtual environments, which offers a chance to deliver health benefits *via* virtual nature (Bohil et al., 2011; Mattila et al., 2020). Additionally, VR can help duplicate a sense of immersion, which is hardly realized *via* traditional two-dimensional (2D) media. According to the findings on actual nature exposure, immersing in natural surroundings may be crucial to receive environmental benefits (Antonelli et al., 2019). Therefore, VR has been deemed as a promising technology when it comes to virtual nature. However, limited studies compared VR with traditional 2D media in simulated nature exposure, which needs extra investigation.

With lower prices and being more portable than before, VR technology has become a practical method for healthcare and rehabilitation (Maggio et al., 2018; Verhoef et al., 2021; Wren et al., 2021). The main types of simulation for virtual environments are real-scene-based 360°videos and computer-generated (CG) scenarios (Yeo et al., 2020). The former can provide a more realistic view, while the latter allows for more interaction with virtual environments. Additionally, many interactive designs have been embedded in virtual environments, such as games and physical activities, which may also enhance the experience of virtual nature. However, it is yet to be concluded whether the benefits of virtual nature may vary with the simulation types and modes of VR. Therefore, we carried out the current review based on published literature to address the following questions:

(1) What benefits can be derived from virtual nature?
(2) Is VR a better medium than traditional media for simulated nature exposure?
(3) Can virtual nature be equivalent to real nature?
(4) Is there a difference between the simulation types and modes of virtual nature?

## Current Virtual Reality Devices for Virtual Nature

Empirically, VR is typically defined in terms of its technological hardware, including computers, head-mounted displays, headphones, and motion-sensing gloves (Steuer, 2010), which offer accessible ways to enable immersion with pleasant virtual environments (Riches et al., 2021). Currently, although multisensory VR has been developed, a head-mounted display (HMD) is still the most common device due to its convenience and affordability. Relevant studies concerning our research questions also only used HMDs to deliver visual and auditory stimuli of nature. In these studies, the virtual environments were displayed *via* 360° videos based on real scenes (360-VR) or CG images generated by computer game engines (CG-VR).

## General Effects of Virtual Nature

**FIGURE 1:**
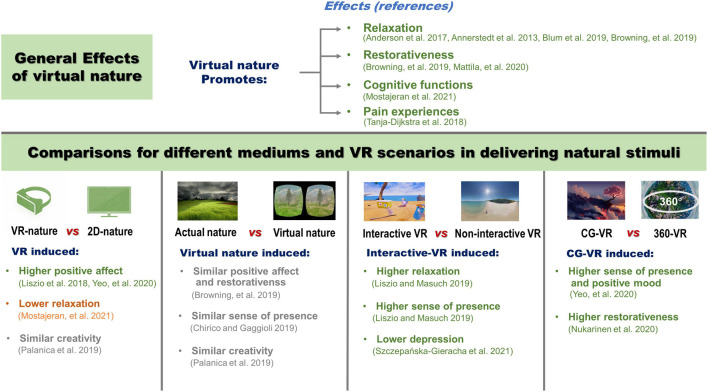
The effects of virtual nature and comparisons for the different mediums and scenarios.

Relaxation is the key function of virtual nature, which includes both psychological and physiological relaxation (Riches et al., 2021). These relaxation effects are usually detected via physiological indices (e.g., heart rate variability, electrodermal activity, and saliva cortisol) and self-reported questionnaires (e.g., the Positive and Negative Affect Schedule and the State-Trait Inventory) (Annerstedt et al., 2013; Anderson et al., 2017; Blum et al., 2019; Browning et al., 2019; Figure 1). Additionally, some studies found that virtual nature may promote restorativeness, which deals with the restoration from attention fatigue (Browning et al., 2019; Mattila et al., 2020). Such a benefit on attention restoration has been traditionally considered as a function of actual nature, especially when it comes to visual stimulation (Ratcliffe et al., 2013; Ohly et al., 2016). However, virtual nature has also been effective, which could be due to its vivid and fascinating views (Mattila et al., 2020). Moreover, there is also evidence for cognition and pain experience. Specifically, a study reported the positive effect of virtual nature on cognitive performance (Mostajeran et al., 2021), which could be related to its restorativeness (Shin, 2011). Tanja-Dijkstra et al. (2018) reported that virtual nature reduced both experienced and recollected pain during simulated pain, which is broadly in line with the recent findings on pain relief and actual nature exposure (Stanhope et al., 2020; Li et al., 2021a). Drawing from previous studies, pain relief could result from both the distraction of VR itself and the audiovisual stimuli of nature (Kline, 2009; Sil et al., 2014; Guo et al., 2015). According to the author, restorative natural stimuli could be more critical.

These benefits from virtual nature are generally consistent with those from actual nature. According to previous studies, visual and audio factors play important roles in obtaining benefits from nature exposure (Kline, 2009; Akers et al., 2012; Wooller et al., 2018; Briki and Majed, 2019). Current VR devices can deliver vivid audiovisual stimuli, and thus, create the illusion of being transported into a virtual world and facilitate presence in a restorative natural environment with the consequent psychological benefits (Smyth et al., 2015). The benefits of nature, such as restorativeness and relaxation, are also regulated by the characteristics of the environment, including environmental quality and structure (Pretty et al., 2005; Gatersleben and Andrews, 2013; Lopez-Pousa et al., 2015). The virtual environments for virtual nature are usually created based on high-quality and pleasant landscapes; thus, they may offer viewers optimized experiences and improve psychological and physiological outcomes (Mattila et al., 2020).

## Virtual Reality vs. Traditional 2D Media

Virtual reality is deemed as a better media than traditional 2D media in delivering audiovisual stimuli. However, there still exist some controversies on the advantages of VR technology (Figure 1). Specifically, two studies found that ocean views being displayed by VR resulted in a higher positive affect than their 2D counterparts (Liszio et al., 2018; Yeo et al., 2020). In contrast, Mostajeran et al. (2021) argued that exposure to a forest in VR is not as effective in reducing stress when compared with photos. Palanica et al. (2019) also reported the similar effects of 2D and VR in enhancing creativity. The inconsistent findings do not support the previous hypothesis that VR may be more effective and beneficial than traditional 2D mediums. Mostajeran et al. (2021) explained that VR may induce an intensive sense of presence, which may be positively correlated with physiological arousal, thus buffering the physiological relaxation of natural stimuli. On the other hand, such psychological and physiological responses may be associated with the properties of virtual nature. For instance, the higher stability of VR videos is found to improve the effect profile of participants and reduce fatigue (Litleskare and Calogiuri, 2019), and the brightness of VR videos may also cause impacts on stress levels during an intervention (Li C. et al., 2020). Besides, the effects may also vary with the demonstrated natural views and the corresponding audio, which may regulate the effectiveness of simulated nature exposure (Annerstedt et al., 2013; Hedblom et al., 2019b; Wang et al., 2019; Lindquist et al., 2020). Therefore, there is still a need to identify beneficial stimuli to optimize the experience of virtual nature.

## Virtual Nature vs. Actual Nature

Relevant studies generally suggested that VR induced nearly equivalent psychological and physiological benefits as real nature (Figure 1). Browning et al. (2019) reported that both virtual nature and actual nature induced similar positive affect and restorativeness. The two other studies further supplemented that a sense of presence and creative thinking were not significantly different between actual and virtual nature (Chirico and Gaggioli, 2019; Palanica et al., 2019). These studies indicate the comparable benefits of exposure to virtual nature and actual nature, which could be due to the vivid audiovisual stimuli discussed above. However, it is not clear why other known beneficial environmental factors did not cause potential differences. For instance, exposure to sunlight, environmental microbiomes, and negative air ions generated by plants were reported to improve emotional outcomes, but these factors are absent in virtual nature (Stanhope et al., 2020). According to Mattila et al. (2020), the absence of other sensory stimuli may make the participants focus on the content of the application, and thus improve psychological outcomes. Additionally, due to the lack of physical engagement, such as walking and contact with things in nature, the brain may process information differently from reality, resulting in some environmental factors being masked or ignored (Taube et al., 2013). Given the limited number of studies that made direct comparisons between the virtual and actual, the differences between the two types of nature exposure still need to be re-examined in the future.

## Interactive Virtual Reality vs. Non-Interactive Virtual Reality

Interactive VR is deemed as a more interesting and motivating tool to promote the engagement of subjects in rehabilitation therapy (Choi and Paik, 2018). Liszio and Masuch (2019) designed two mini-games (throwing a coconut and flower watering) at a virtual beach and found that interactive VR elicited a higher sense of spatial presence and led to a higher heart rate variability level, indicating better physiological relaxation. Likewise, Szczepańska-Gieracha et al. (2021) designed a 4-week VR treatment containing interactive games (simulated plant watering and a coloring task) and observed reduced depression in an elderly population, which brings a practical indication of using virtual nature to improve the health of this special population. However, another study using an active VR intervention failed to identify its advantage over a normal VR experience (Tanja-Dijkstra et al., 2018). This might be due to the different designs of interactivity, for the former two studies used mini-games, while Tanja-Dijkstra et al. (2018) used a controller for participants to manipulate the virtual environment, which might demand less attention, and thus, less engagement. According to existing evidence, the game design could be relevant to the health benefits of interactive VR.

## 360-VR vs. CG-VR

Two formats were used to express virtual nature in the relevant studies, namely, 360° videos and CG scenarios. However, only two studies investigated the difference between the two forms (Figure 1). Yeo et al. (2020) reported that CG-VR induced a significantly greater sense of presence and positive mood than 360-VR, but boredom remained similar in the two conditions. However, although Yeo et al. (2020) have demonstrated similar views on the marine world in both conditions, the images are not completely consistent. To make a fair comparison, Nukarinen et al. (2020) constructed a CG scenario and made a 360-VR based on the same view of actual nature. Nukarinen et al. (2020) reported that the CG-VR was more emotionally restorative than the 360-VR, which showed the advantage of CG-VR over 360-VR in simulated nature exposure.

## Limitations of Virtual Nature

Though VR is an advanced form of media to access nature, the general VR applications rely on audiovisual stimuli and do not exploit the addition of other sensory stimuli (Melo et al., 2020). Recent evidence indicates that VR with multisensory stimuli may elicit a positive impact on users (Melo et al., 2020). However, only a few studies employed multisensory VR to focus on the effects of virtual nature, and only the olfactory stimulus was considered, while other popular sensory stimuli such as haptics were not included (Hedblom et al., 2019a; Sabiniewicz et al., 2021). According to Hedblom et al. (2019a), the olfactory stimuli of nature may be better at decreasing stress than visual stimuli, which could be related to the odor of trees (Ikei et al., 2015). However, due to limited devices (HMD only), none of the multisensory VR applications has been adopted in the studies concerning our research questions.

On the other hand, cybersickness is a known problem of VR experience, but it received insufficient concern (Martirosov et al., 2021). Only two studies concerning our research questions investigated the conditions of cybersickness (Liszio and Masuch, 2019; Mostajeran et al., 2021), but no method was adopted to avoid this issue, and the effects of cybersickness were not investigated either. Therefore, it is still unclear whether the benefits of virtual nature can be reduced by negative symptoms and the extent of their effect. Also, it cannot be determined whether the different levels of immersion or types of environments play a role in inducing cybersickness. These uncertainties will bring difficulties in generalizing the known benefits of virtual nature to the public (Mostajeran et al., 2021). According to Litleskare and Calogiuri (2019), increasing the stability of the camera may reduce the symptoms of cybersickness in virtual nature, which should be considered in future studies.

## Direction for Future Study

### Application of Virtual Nature

Virtual nature is deemed as a potential method for relaxation (Riches et al., 2021). Theoretically, patients and the elderly are most likely to benefit from the technology due to the inconvenience of physical activity. However, the current studies mainly aimed to explore the effects of virtual nature, without paying much attention to special populations. For instance, only two studies concerning our research questions focused on patients and the elderly (Lakhani et al., 2020; Szczepańska-Gieracha et al., 2021). Therefore, future studies need to involve more special populations, such as people who are quarantined due to COVID-19, to explore more practical and useful interventions. Additionally, as most studies are concentrated on the acute effects of simulated nature exposure, long-term interventions are needed to check if virtual nature can be beneficial without the sense of novelty (Riches et al., 2021).

### Simulated Green Exercise

Green exercise is a concept of nature-based exercise referring to exercises carried out in natural environments, which aims for the combined health benefits of nature exposure and physical activities (Mnich et al., 2019). Due to the known health and training benefits of green exercise, many attempts have been made to replicate green exercise *via* media, and evidence indicates that a simulated natural environment may help reduce perceived exertion and provide a smooth exercise experience (Akers et al., 2012; Li et al., 2021b). As a technology that creates a better sense of immersion, VR is reported to have positive effects on the presence and perceived environmental restorativeness during a simulated nature walk, which is equivalent to the actual nature walk counterpart (Calogiuri et al., 2017). However, no benefit in mood was found during high-intensity interval cycling while viewing a virtual natural scene, indicating that the benefits of simulated green exercise may vary with the measured dimension and the type of exercise (Alkahtani et al., 2019). Some studies have revealed the effects of VR on enhancing exercise experience and training outcomes (Cho et al., 2016; Wender et al., 2019; Qian et al., 2020). However, the role of the demonstrated virtual environment received less attention, and the benefits of simulated green exercise need to be investigated further.

### Blue vs. Green Virtual Environment

The virtual natural environments demonstrated in the relevant studies contained both blue and green environments (see Supplementary Table 1). A recent study has implied the difference between actual green and blue environments (White et al., 2021). According to Wang et al. (2019), the corresponding responses may also vary with the type of virtual environment. Although considerable studies have focused on the differences between virtual nature and a virtual urban environment (Valtchanov and Ellard, 2010; Yu et al., 2018; Palanica et al., 2019; Mostajeran et al., 2021), there is a lack of understanding of the difference between green and blue virtual natural environments, which remains a topic for future studies.

## Conclusion

The current review summarized the benefits and effectiveness of nature exposure *via* VR technology. The existing evidence generally supports that virtual nature may induce a relaxation effect and also benefit attentional resources, cognitive performance, and pain experience. Game designs may be useful in creating interactive VR scenarios that may improve the virtual experience. Additionally, CG scenarios may be more effective than 360° videos in inducing the psychological benefits of virtual nature. These findings indicate the potential role of simulating nature exposure in health promotion in urban and certain special populations. According to the interests of relevant research fields, the application of virtual nature, simulated green exercise, and diverse virtual natural environments may be topics for further study.

## Author Contributions

HSL wrote the manuscript. ZY, XZ, HW, and HWL revised the manuscript. YC and GZ supervised the project and amended the final version of the manuscript. All authors contributed to the article and approved the submitted version.

## Conflict of Interest

The authors declare that the research was conducted in the absence of any commercial or financial relationships that could be construed as a potential conflict of interest.

## Publisher’s Note

All claims expressed in this article are solely those of the authors and do not necessarily represent those of their affiliated organizations, or those of the publisher, the editors and the reviewers. Any product that may be evaluated in this article, or claim that may be made by its manufacturer, is not guaranteed or endorsed by the publisher.
