# Supplementary material for: Access to Nature via Virtual Reality: A Mini-Review
Source: Front Psychol. 2021 Oct 5;12:725288. doi: 10.3389/fpsyg.2021.725288 (PMC8523668; doi:10.3389/fpsyg.2021.725288)
Supplement: Supplementary file 1 [file Table_1.DOCX]

Supplementary Material

**Supplementary Table 1. Characteristics of the relevant studies concerning our research questions**

| **Study** | **Experimental design** | **Participants** | **Environment type** | **Environment properties** | **Stress induction before VR** | **Experimental group** | **Control**  **group** | **Duration for single exposure** | **Measurements** | **Main findings** |
| --- | --- | --- | --- | --- | --- | --- | --- | --- | --- | --- |
| Anderson et al. (2017) | Crossover-RCT | 18 (9 females)  32±12 year | Blue | 360-VR | Yes | VR (natural scene) | VR (control scene was comprised of empty indoor classrooms) | 15 min | 1. Value of VR Questionnaire (VVR)  2. Electrodermal activity (EDA)  3. Electrocardiogram (EKG)  4. Heart rate variability (HRV)  4. The positive and Negative Affect Schedule (PANAS)  5. The 15-question Modified Reality Judgment and Presence Questionnaire (MRJPQ) | Virtual nature reduced EDA and negative affect while increased MRJPQ scores. |
| Annerstedt et al. (2013) | RCT | 30 (male only)  27.7±6.7 year | Green | CG-VR | Yes | VR (forest with sounds OR forest without sounds) | No VR | 15 min | 1. Spielberger state and trait anxiety inventory (STAI-S)  2. Saliva cortisol  3. Heart rate variability (HRV)  4. T-wave amplitude | Parasympathetic activation was found in the group subjected to sounds of nature in a virtual natural environment. |
| Blum et al. (2019) | RCT | 60 | Blue | CG-VR | Yes | VR (beach scenery at sunset) | No VR | 10 min | 1. State version of the State Trait Anxiety Inventory (STAI-S)  2. Relaxation self-efficacy  3. Cognitive Interference Questionnaire (CIQ)  4. State Mindfulness Scale (SMS)  5. Computerized modified Stroop task  6. Heart rate variability (HRV) | Virtual nature buffered perceived stress in the subsequent stressor task, increased relaxation self-efficacy, reduced mind wandering, helped participants focus on the present moment, and helped preserve attentional resources. |
| Browning et al. (2019) | RCT | 82 (39 females)  20±1.2 year | Green | 360-VR | No | VR (natural scene) | Real nature or No VR | 6 min | 1. Positive and Negative Affect Schedule (PANAS) scale  2. Perceived Restorativeness  Scale (PRS)  3. Skin conductance levels (SCL)  4. Natural Beauty Subscale of the Engagement with Beauty Scale (EBS) | Both types of nature exposure increase physiological arousal, benefit positive mood levels, and are restorative compared to an indoor setting without nature; outdoor exposure increased positive mood levels but virtual nature stay the same. |
| Chirico and Gaggioli (2019) | RCT | 50 (25 females)  23.78±3.01 years | Blue and green | 360-VR | No | VR (natural scene) | Real nature | 5 min | 1. state Positive and Negative Affect scale (PANAS)  2. ITC-Sense of Presence Inventory (ITC-SOPI)  3. 8-items emotion scale | Emotions elicited by virtual and natural conditions were not significantly different. The only exception was anger, which was significantly higher in the natural condition, and amusement, which was  significantly higher in the virtual condition. Sense of physical presence and engagement dimensions of presence  did not significantly differ between virtual and real conditions. |
| Lakhani et al. (2020) | RCT | 24 with spinal cord injury | n.m. | n.m. | no | VR and regular rehabilitation | Regular rehabilitation | 20 min | 1. Patient Health Questionnaire-8 (PHQ-8)  2. Adapted versions of the Depression Intensity  Scale Circles | Levels of happiness, relaxation, and feeling good, were significantly higher subsequent to engaging with each VR session. Between-group differences in PHQ-8 scores were significantly greater for participants who experienced the intervention during the first week compared to participants within the control group. |
| Mattila et al. (2020) | Pre-Post | 100 (44 female) | Green | CG-VR | No | VR (virtual forest) | | 5 min | 1. Subjective Vitality Scale (SVS)  2. Positive and Negative Affect Scale (PANAS)  3. Restoration Outcome Scale (ROS) | The VR environment was generally perceived as restorative as the actual forest environments, and more fascinating and coherent. |
| Mostajeran et al. (2021) | Crossover-RCT | 34 (11 females)  27.26±4.14 years | Green | 360-VR | Yes | VR or Picture (Forest or urban scenes) | VR (blank scene) | 6 min | 1. State Trait Anxiety Depression Inventory-State (STADI-S)  2. Profles of Mood States (POMS)  3. Short Stress State Questionnaire (SSSQ)  4. Perceived Stress Scale (PSS)  5. Igroup Presence Questionnaire (IPQ)  6. Simulator Sickness Questionnaire (SSQ)  7. Galvanic skin response (GSR)  8. Heart rate | Virtual forest environment had a positive effect on cognition and the urban environment disturbed mood regardless of the mode of presentation. In addition, photos of either urban or forest environment were both more effective in reducing physiological arousal compared to 360-VR |
| Nukarinen et al. (2020) | RCT | 24  26 years | Blue | 360-VR + CG-VR | No | VR (360-video or C.G.) | Real nature | 10 min | 1. Heart rate (HR)  2. Heart rate variability (HRV)  3. Electrodermal activity (EDA)  4. Positive and negative affect scale (PANAS) | Some of the benefits of the real forest could also be obtained using virtual equivalents. Furthermore, the 3D forest may be emotionally more restorative than the 360-degree video forest. |
| Palanica et al. (2019) | RCT | 84 and 97 | Green + Blue | 360-VR | No | Experiment 1: 2D vs. VR  Experiment 2: 2D vs. Real nature | | 4 min | 1. Creativity | Experiment 1 showed that nature videos facilitated higher creativity compared to urban videos, across mediums of viewing stimuli through a 2D mobile tablet and a 3D VR headset. Experiment 2 suggested that both actual nature and urban conditions evoked the same relatively high level of creativity. |
| Liszio et al. (2018) | RCT | 62 (36 females)  22.6±5.36 | Blue | n.m. | Yes | VR or Desktop screen or No media | | 7 min | 1. Heart Rate Variability (HRV)  2. Salivary Cortisol  3. State-Trait Anxiety  Inventory (STAI)  4. Positive and Negative Affect Scale (PANAS) | Systematic changes in physiological (heart rate variability, cortisol) and  psychological (anxiety, affect) measures were observed: The VR group experienced significantly lower stress and higher positive affect than the desktop group and no media control. |
| Liszio and Masuch (2019) | RCT | 41 (25 females)  23.7±5.67 years | Blue | CG-VR | Yes | VR (interactive or non-interactive scenarios) | No VR | 9 min | 1. State-Trait Anxiety  Inventory (STAI)  2. Positive and Negative Affect Scale (PANAS)  3. Heart rate variability (HRV) | Relaxation and positive affect were significantly increased in the interactive VR condition compared to the non-interactive and the control group. The experience of spatial presence in the VR  app is a significant predictor of positive feelings during the stress phase. |
| Szczepańska-Gieracha et al. (2021) | RCT | 25 (females only)  70.73 years | Green | CG-VR | No | VR (interactive or non-interactive scenarios) | Not involved | 20 min | 1. 30-iterm Geriatric Depression Scale (GDS-30)  2. Stress Questionnaire (PSQ)  3. Hospital Anxiety and Depression Scale (HADS). | In the VR group, the GDS-30 score was reduced by 36%, and the result persisted in the follow-up tests. Immersive virtual therapy significantly lowered the intensity of depressive symptoms, as well as stress and anxiety levels in older women taking part in the group-based multimodal therapeutic programme, whose earlier therapy had not brought thee expected results. |
| Tanja-Dijkstra et al. (2018) | RCT | 85 (51 female)  21.72±4.67 years  70 (>18 years) | Blue | CG-VR | Yes | VR (interactive or non-interactive scenarios) | No VR | 4 min | 1. McGill Pain Questionnaire (SF-MPQ)  2. the Modified Dental  Anxiety Scale (MDAS) | Virtual nature reduced both experienced and recollected pain compared with no VR. |
| Yeo et al. (2020) | RCT | 96 (>18 years) | Blue | 360-VR | Yes (boredom) | TV or CG-VR or 360-VR | | 5 min | 1. Presence and Reality Judgement Questionnaire  2. Positive and Negative Experiences scale (SPANE)  3. Nature connectedness | Virtual nature reduced boredom and negative affect and increased positive affect and nature connectedness. Although reductions in boredom and negative affect were similar across all three conditions, CG-VR was associated with significantly greater improvements in positive affect than TV, which were mediated by greater experienced presence and increases in nature connectedness. |

**Notes:** n.m. indicates not mentioned; CG-VR or 360-VR indicates VR intervention using CG scenarios or 360-video.
